# Hemorrhage*Read before the Louisville Society of Medicine, December 5, 1898.

**Published:** 1899-03

**Authors:** A. H. Falconer


### Selections and Abstracts.

---

### HEMORRHAGE.*

By A. H. FALCONER, M.D.

Hemorrhage may arise from wounds of arteries, veins, or capillaries, or from wounds of the three combined. In arterial hemorrhage the blood is a scarlet red and appears in jets from the approximal end of the vessel; these jets are synchronous with the pulse-beat. The stream never intermits; the stream from the distal end is darker and not pulsatile. Venous hemorrhage is denoted by a dark hue of blood and its continuous stream. In capillary hemorrhage red blood wells up like water from a sponge. If an artery ruptures in an extremity, there is no pulsation below rupture; if a vein ruptures in an extremity, intense edema occurs; profuse hemorrhage induces constitutional symptoms, and death may occur in a few seconds. Generally after the bleeding has gone on for some time, syncope occurs, which is nature's effort to arrest hemorrhage, for duing this time the feeble circulation and the increased coagulability of the blood give time for the formation of a clot. When reaction occurs, the clot may hold or it may be washed away with a renewal of bleeding and syncope; this may be repeated until death occurs.

After a profuse hemorrhage one is intensely pale and of sort of greenish tinge, the eyes are fixed in a glassy stare and pupils widely dilated, respirations are shallow and sighing, skin covered with a cold sweat, legs and arms extremely cold, pulse small, soft, compressible, fluttering or often can not be detected at all, heart very weak. When such a dangerous condition is due to a visible hemorrhage, temporarily arrest hemorrhage by digital pressure in the wound, lower the head, and make compression on the femorals and subclavians so as to divert more blood to the brain, apply artificial heat, inject hypo., brandy, and strychnia ($\frac{1}{30}$ gr.), and as soon as reaction begins, arrest hemorrhage permanently by ligature.

Hemostatics used are (1) the ligature, (2) acupressure, (3) torsion, (4) compression, (5) styptics, (6) the actual cautery, (7) forced flexion of the limbs. The ligatures may be made of silk, catgut, etc., but they must be made aseptic. The ligatures should be about ten inches long; the vessel is drawn out with forceps and separated from surrounding tissues. Some use tenaculum to catch the vessel with, but forceps are best in most cases, because the tenaculum makes a hole through which

---

*Read before the Louisville Society of Medicine, December 5, 1898.

blood may exude. Tenaculum best used when vessels lie in hard tissues; tie with a reef knot both ends of the vessel. If an artery is incompletely divided, tie on each side of the cut and entirely sever the vessel between the ligatures. If bleeding comes from an artery very close to its point of origin, tie the main trunk as well as the bleeding branch, otherwise the clot will be too short and secondary hemorrhage will be inevitable. Never include a nerve in ligating.

By means of torsion the internal and middle coats are ruptured and the external coats twisted. It is a safe procedure, and is practiced by many surgeons of high standing upon vessels as large as the femorals.

Acupressure is pressure with a pin passed under a vessel (transfixion), leaving a little tissue on each side between the pin and the vessel. A needle can be passed under a vessel and a wire thrown over the vessel and twisted (circumclusion); the needle can be inserted on one side, passed through half an inch of tissue up to the vessel, be given a quarter twist, and be driven in the tissues across the artery (torsoclusion); some tissue is picked up on the needle, folded over the vessel, and pinned to the other side (retroclusion). Acupression is used for inflamed or atheromatous vessels, in sloughing wounds, and where a ligature will not hold.

Compression is either direct or indirect; that is, in the wound or upon its artery of supply; compression and hot water, 120° F., will stop capillary bleeding, and also that from superficial veins. The knotted bandage of the scalp will arrest bleeding from the temporal artery; long-continued pressure causes pain and inflammation. Chemicals are now rarely used. In epistaxis we may pack with plugs of gauze saturated with antipyrine. Bleeding from a tooth-socket, pack with styptic cotton, also in an incised urinary meatus.

Cold water or ice acts as a styptic by producing reflex vascular contraction. Hot water produces contraction and coagulates albumin; the temperature should be 115° to 120° F. A mixture of equal parts of alcohol and water will often check capillary oozing.

The actual cautery is a very ancient hemostatic; it is still used in bleeding after removal of malignant growths, in continued hemorrhage from the prostatic plexus of veins, and to stop oozing after the excision of venereal warts. We are driven to it in the "bleeders," that is, those persons who have a hemorrhage diathesis, and who may die from having a tooth pulled or from a scratch, etc.

Forced flexion is a variety of indirect compression; it will stop bleeding, but will soon become intensely painful. If we fail to look into a wound, we can not know what is cut; it may be only a branch and not a main trunk. Ligate veins as you would arteries; in a wound of the superficial palmer arch tie both ends of the divided vessel. In a wound of the deep palmer arch, enlarge the wound if necessary in the direction of the flexor tendons, at the same time maintaining pressure on the brachial artery; if the artery can be caught by but can not be tied over the point of forceps, leave the forceps on for four days. If vessel can not be caught by forceps or tenaculum, insert a small piece of gauze in the depth of the wound, over this a larger piece, and keep adding over this bit after bit, each one larger than the one before, until there is a conical pad, the apex of which is against the extremities of the cut arch and the base well external to the palm; bandage each finger and thumb, put a piece of metal over the pad, also a compress in front of the elbow, flex the forearm upon the arm, wrap the hand in gauze, place the arm upon a straight splint, apply firmly an ascending spiral reverse bandage of the arm, and hang the arm in a sling. The pad is left in place for six or seven days unless bleeding keeps up or recurs. If bleeding begins again, ligate the radial and ulnar. If this fails, we know that the interosseous artery is furnishing the blood, and the brachial must be tied at the bend of the elbow; if this fails, amputate the hand. In primary hemorrhage, if the bleeding ceases do not disturb the parts to look for the vessel; if the vessel is clearly seen in the wound, tie it; otherwise, do not, as the hemorrhage may not recur.

When a man has delirium tremens, mania, or when he is a heavy drinker, in these cases, always look for the vessel and tie it. When a person is bleeding to death, arrest hemorrhage temporarily by digital pressure in the wound, and apply above the wound a tourniquet or Esmarch bandage. Bring about reaction, then ligate, but do not operate during collapse if the bleeding can be controlled by pressure. When a branch of a large vein is torn close to the main trunk, tie the branch and not the main trunk; apply practically a lateral ligature. If, after tying the cardial extremity of a cut artery, the distal extremity can not be found, even after a careful search, enlarge the wound and firmly pack it. In bleeding from the internal mammary artery pass a large curved needle holding a piece of silk into the chest under the vessel and out again, then tie the thread tightly. In collapse due to the puncture of a deep vessel, the bleeding having ceased, do not hurry reaction by stimulants; give the clot a chance to hold; wrap the patient in hot blankets if the condition is dangerous; however, stimulate to save life. In punctured wounds, as a rule, try pressure before using ligature.

After a severe hemorrhage always put the patient to bed and elevate the damaged part if it be an extremity or the head. A clot which holds for twelve hours after a primary hemorrhage will probably hold permanently, but even after twelve hours be watchful and insist on rest. In bleeding from a tooth-socket use ice; if this fails, plug with gauze infiltrated with tannin; close the jaws upon the plug, and hold them with Barton's bandage. If this fails, soak plug in Monsel's solution, and lastly use cautery. Pressure on the carotid and ice over the jaw and neck are indicated; it may be necessary to tie the common carotid. A ruptured varicose vein requires a compress bandage from the periphery up and the limb elevated. Pressure above a wound stops arterial hemorrhage, but aggravates venous bleeding. In severe bleeding from the ear, elevate the head and put on ice-bag over the mastoid; give opium and lead acetate, and if blood runs in the mouth, plug the eustachian tube with a piece of catheter.

Subcutaneous hemorrhage demands that an incision be made and ligation be performed. Bleeding from a cut urethral meatus requires the insertion of styptic cotton and application of pressure. Moderate bleeding from the urethra can usually be arrested by a hot bougie or hot injections; ice to the perineum does good; if these means fail, perform an external urethrotomy and reach the bleeding point.

Vaginal hemorrhage requires the tampon or the ligature. Bleeding from the stomach is treated by the swallowing of ice, giving tannic acid, dose twenty to thirty grains. Never give tannic acid and Monsel's solution at the same time, as they mix and form ink. Opium is usually ordered; acetate of lead, opium and gallic acid are favorite remedies, and ergot is sometimes used; give no food at all. Hemorrhage from phthisis or bleeding from the lungs is treated by morphia hypo., by perfect rest, dry cups or ice over the affected spot if it can be located, by ergot and gallic acid; gallic acid aids coagulation.

Recurrent hemorrhage, called also consecutive, intermediate or intercurrent, comes on during reaction from an accident or operation; that is, during the first forty-eight hours, and s usually due to a badly applied ligature, or it may result from vascular excitement, or from hypertrophied heart, the jumping artery loosing the ligature. The Esmarch apparatus is not unusally the cause. To lessen the danger of the Esmarch apparatus, use a broad constricting band rather than a tube. In any severe recurrent hemorrhage, open up wound at once and ligate.

Secondary hemorrhage may occur at any time in the period of forty-eight hours after the accident or operation and the complete cicatrization of the wound. Secondary hemorrhage may be due to atheroma, to slipping of ligature, to the inclusion of a nerve, fascia or muscle in the ligature, to sloughing, erysipelas, septicemia, pyemia, gangrene, and to overaction of the heart. If during an operation the vessels are found atheromatous, acupressure had best be used, or pass a thread by means of a curved needle around the vessel, including a cushion of tissue in the loop of the ligature to prevent cutting through the vessel. One great trouble with atheromatus arteries is that their coats can not retract; another trouble is that the ligature cuts entirely through them. If after an operation the pulse is found to be forcible, rapid, and jerking, give aconite, opium, and low diet.

Hemorrhage from the prostate may follow the relief of retention of urine, may be due to stone, inflammation, tumors, etc., or may arise from traumatism, instrumental or otherwise. The color of the urine is usually bright red, but if long retained in the bladder it becomes black and often tarry; the reaction is alkaline; the clots when floated out are large and without definite shape. In micturition the urine is clear or only a little colored at the beginning, but becomes darker and darker as micturition ends, at which time the flow may consist of almost pure blood. In very small vesical hemorrhage the urine may be smoky; the microscope shows colorless and swollen corpuscles and many polygonal cells. In urethral hemorrhage, blood comes independently of micturition, or blood comes out first, and is followed by pure water. Urethral hemorrhage arises from an acute urethritis, from an inflamed stricture, from the passage of an instrument, or from some other traumatism. Intracranial hemorrhage may be either spontaneous or traumatic; in the vast majority of instances spontaneous hemorrhage comes from the lenticulo-striate artery and produces apoplexy. Traumatism during delivery is a not unusual cause of hemorrhage from the middle meningeal artery.

A traumatic hemorrhage may take place (1) between the bone and the dura (extra dural), (2) between the dura and brain (subdural), (3) in the brain substance (cerebral). Extra-dural hemorrhage arises from the middle meningeal, or more often from one of its branches; it is usually but not always accompanied by fracture; in fact, in some cases not even a bruise can be found. The accident may or may not cause temporary unconsciousness, but even if it does, from this unconsciousness the patient almost always reacts, and there is a distinct period of consciousness between the accident and the lasting coma, the coma being due to a pressure from a continually increasing mass of extravasated blood. If the main trunk or a large branch is ruptured, the period of consciousness is short; if a small branch is ruptured, the period of consciousness is prolonged for hours, or perhaps for days. The pulse becomes frequent, the breathing stertorous, the temperature rises, and in compound fractures the pressure of the escaping blood may force brain matter out of the wound. In treating extra-dural hemorrhage, localize the clot not by the seat of the wound or contusion but by the symptoms entirely, and trephine to find the bleeding vessel.

Subdural hemorrhage is usually due to depressed fracture and rupture of the middle cerebral artery, or of a number of small vessels; the symptoms are identical with those of extra-dural bleeding. The treatment is trephining at the first hemorrhagic point, turning out the clot, ligating the bleeding point, elevating any depression of bone, and draining and stitching the dura with catgut.

Rupture of a sinus usually arises from compound fractures or during a brain operation. The treatment, if the rupture happens from a fracture, is trephining, enlarging the opening, and pack with one large piece of iodoform gauze, or catch the rent with hemostatic forceps, leaving them in place for three or four days, or apply a lateral ligature and elevate depressed bone. In rupture during operation, control hemorrhage by packing. In prolonged hemorrhage from leech-bite, try compression over a plug saturated with alum or tannin. If this fails, pass under the wound a hair-lip pin and encircle it with a piece of silk; if this fails, use the actual cautery. Umbilical hemorrhage in infants requires pressure over a plug containing tannin, alum or gelatin solution. If compression fails, pass hair-lip pins under the navel and apply a twisted suture. If this fails, use the actual cautery.

Rectal hemorrhage requires elevation of the buttocks, insertion of plugs of ice, ice to the anus and perineum, astringent injections (alum), and the internal use of opium and acetate of lead. If these means fail, plug the bowel over a catheter, or insert and inflate a Peterson bag or colpeurynter, or tampon and use a T bandage. If the bleeding persists, or if a considerable vessel is bleeding, stretch the sphincter, catch the bowel and draw it down, seize the vessel and tie it if possible; if not, leave the forceps in place. Failing in this, the actual cautery must be used. Vesical hemorrhage usually ceases spontaneously, in which case the urine must be drawn off and the viscus be washed out frequently with a solution of boric acid to prevent septic cystitis. If blood-clots prevent the flow of urine, break them up with a catheter or lithotrite and inject vinegar and water, a two-per-cent solution of carbolic acid, or a solution of bicarbonate of sodium. Perfect quiet is to be maintained, cold acid drinks to be given, ice-bags to be put to the perineum and hypogastric region, and opium with acetate of lead, ergot or gallic acid to be given by the mouth. If the hemorrhage is severe or persistent, perform a suprapubic cystotomy. Renal bleeding requires ice to the loin, tannic acid and opium, gallic and sulphuric acid, and perfect quiet. If the bleeding threatens life and the diseased organ is identified, make a lumbar incision and suture or perform a nephrectomy; if not sure which organ is diseased, perform an abdominal nephrectomy. The use of a cystoscope will show from which ureter blood is emerging.

In hemorrhage from the small bowel, give acetate of lead and opium, sulphuric acid, or Monsel's salt in pill form (three grains), allow no food for a time, and insist on a liquid diet for a considerable period. If hemorrhage threatens life, do a celiotomy and find the cause; if ulcer exists, excise it. If violent hemorrhage follows injury, explore to discover the cause. In bleeding from the large bowel, use styptic injections (ten grains of alum or five grains of bluestone to one ounce of water). If bleeding is low down, use small amounts of solution; if high up, large amounts. Do not use absorbable poisons. In dangerous cases perform an exploratory operation to find the cause.

Severe uterine hemorrhage (unconnected with pregnancy) requires the tampon. Persistent hemorrhage due to morbid growths, may require removal of the tubes and appendages, ligation of the uterine and ovarian arteries, or hysterectomy. Post-partum hemorrhage is often controlled by ergot, hot injections, elevation of hips, the introduction of a hand with or without ice, ice over the abdomen, etc.—*American Practitioner and News.*

